# Supplementary material for: The Adverse Effects of TiO2 Photocatalycity on Paraloid B72 Hybrid Stone Relics Protective Coating Aging Behaviors under UV Irradiation
Source: Polymers (Basel). 2021 Jan 14;13(2):262. doi: 10.3390/polym13020262 (PMC7830140; doi:10.3390/polym13020262)
Supplement: Supplementary file 1 [file polymers-13-00262-s001.pdf]

Article

The Adverse Effects of TiO_2 Photocatalycity on Paraloid B72 Hybrid Stone Relics Protective Coating Aging Behaviors under UV Irradiation

Wenjuan Li [1,*], Junling Lin [1], Yaru Zhao [1] and Zihe Pan [2,*]

[1] College of Art, Taiyuan University of Technology, 209 University Avenue, Jinzhong 030600, China; junling_lin98@163.com (J.L.); zhaoyaru123rhea@163.com (Y.Z.)

[2] Institute of Resources and Environmental Engineering, Shanxi University, 92 Wucheng Road, Taiyuan 030006, China

* Correspondence: liwenjuan@tyut.edu.cn (W.L.); panzh@sxu.edu.cn (Z.P.)

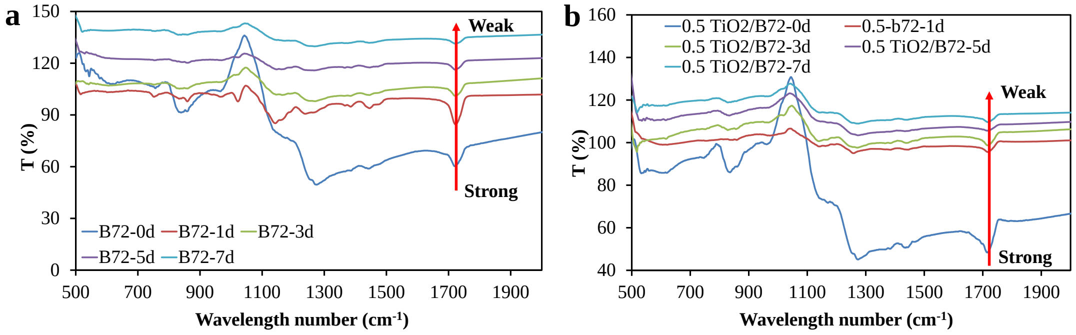

Figure S1. (**a**) and (**b**) are the FTIR spectrum of B72 and 0.5 TiO_2/B72 coatings after different UV irradiation time.
